# The Pupil Knows: Pupil Dilation Indexes and Their Inhibitory Ability in Normal Aging

**DOI:** 10.3390/jcm12144778

**Published:** 2023-07-19

**Authors:** Mohamad El Haj, Claire Boutoleau-Bretonnière, Guillaume Chapelet

**Affiliations:** 1 Institut Universitaire de France, 75000 Paris, France; 2 CHU Nantes, Clinical Gerontology Department, Bd Jacques Monod, 44093 Nantes, France; guillaume.chapelet@chu-nantes.fr; 3 LPPL–Laboratoire de Psychologie des Pays de la Loire, Faculté de Psychologie, Université de Nantes, Chemin de la Censive du Tertre, BP 81227, Cedex 3, 44312 Nantes, France; 4 CHU Nantes, Inserm CIC04, Département de Neurologie, Centre Mémoire de Ressources et Recherche, 44000 Nantes, France; claire.boutoleau-bretonniere@univ-nantes.fr; 5 Inserm, TENS, The Enteric Nervous System in Gut and Brain Diseases, Université de Nantes, 44000 Nantes, France

**Keywords:** aging, inhibition, pupil, pupil size, pupillometry

## Abstract

Pupil dilation is considered an index of cognitive effort, as the pupil typically dilates as the cognitive load increases. In this paper, we evaluated whether older adults demonstrate increased pupil size when performing tasks requiring cognitive inhibition. We invited 44 older and 44 younger adults to perform the Stroop task while their pupil dilation was recorded with eye-tracking glasses. The dependent variables were the number of accurate responses on the Stroop task as well as pupil size in the three conditions of the task (i.e., color naming, word reading, and the interference condition). The results demonstrated less accurate responses in the interference condition than in the color-naming or word-reading conditions, in both older and younger adults. Critically, larger pupil dilation was observed in the interference condition than in the color-naming and word-reading conditions, in both older and younger adults. This study demonstrates that pupil dilation responds to cognitive effort in normal aging, at least in the interference condition of the Stroop task.

## 1. Introduction

Cognitive aging has been widely associated with decreased inhibitory processes. According to the inhibition hypothesis, aging triggers difficulties in suppressing or ignoring irrelevant information [1,2]. Research has demonstrated difficulties in inhibiting irrelevant information in older adults [3,4]. At a neurological level, age-related inhibitory decline has been associated with changes in the frontal lobes in structures supporting attentional control, such as the dorsolateral prefrontal and parietal cortices [5]. Age-related changes can be also observed in the pupil. The pupil size decreases with aging (i.e., miosis) [6,7], which can be attributed to degeneration of the dilation muscle in the iris [6,7]. While pupil dilation has been studied using the Stroop task in younger adults [8,9,10,11,12], it is not the case in older adults. In this study, we propose an original, non-invasive physiological evaluation of age-related inhibitory changes, investigating whether the performance of older adults in the Stroop task is associated with changes in pupillary responses. 

Inhibition can be evaluated using the Stroop task, in which participants are typically presented with written words appearing in various colors (e.g., the word ‘blue’ written in the color red) and they are required to inhibit reading the word (blue) and only name the color (red). The Stroop task is considered a reliable measure of inhibitory control, i.e., the ability to suppress irrelevant information and resist interference. Performances on the Stroop task have been found to induce pupil dilation and research has consistently reported larger dilation in younger adults in incongruent trials than in congruent ones [8,9,10,11,12]. Laeng et al. [11] recorded the pupil activity of participants (*M* age = 26.8 years) using the Stroop task and found that pupil size increased as the task demands increased. These findings converge with a wealth of other findings showing how pupil dilation increases with cognitive load, as demonstrated in the seminal paper studies of Hess and Polt [13,14] and later Kahneman and Beatty [15]. In these studies, participants were asked to memorize a series of digits, with the length of the digit sequences gradually increasing throughout the test. The results demonstrated increased pupil size with the increased number of digits successfully retained. These findings have been consistently replicated in subsequent studies, indicating that pupil size increases with each additional digit retained in digit span tasks, reaching its peak when the length of the digits surpasses the capacity of working memory [16,17,18,19,20]. The effect of cognitive load on pupil dilation has been reported by other research, demonstrating how this dilation increases during processing of complex vs. simple sentences [21], or during complex vs. simple visual search [22]. Pupil dilation may thus be considered as a reliable marker of cognitive load. Increased pupil dilation has been reported in the interference condition of the Stroop task in younger adults, which may be attributed to the high cognitive load of this condition. 

While research has investigated pupil dilation in the Stroop task [8,9,10,11,12], it has not examined the effect of aging. We thus assessed whether older adults would show increased pupil dilation when performing the interference condition of the Stroop task compared with the control conditions (i.e., color-naming and word-reading conditions). We were also inspired by research assessing the effects of cognitive load on pupil dilation in aging. For instance, Piquado, Isaacowitz [23] recorded pupil dilation in older and younger adults while processing digit lists that varied in length (Experiment 1) or while processing sentences that varied in both syntactic complexity and length. Piquado, Isaacowitz [23] applied a normalized measure of pupil size to overcome the limited range of pupil dilation typically observed in normal aging (i.e., compared with younger adults, older adults tend to demonstrate smaller pupil dilation in response to ambient light). Thanks to this correction, Piquado, Isaacowitz [23] observed that pupil size increased with the length of digits (Experiment 1) and sentences (Experiment 2) in both populations. These findings are important as they may account for the lack of pupil dilation as a function of memory load reported in a study by Van Gerven, Paas [24]. These authors recorded pupil dilation in older and younger adults during a memory-search task, which involved different levels of memory load. They reported that pupil dilation was not sensitive to memory load in older adults, whereas it increased as a function of this load in younger adults. The lack of pupil reaction in older adults reported by Van Gerven, Paas [24] may be attributed to the lack of a normalized measure of pupil size. In our study, we applied a normalized measure to overcome the limited range of pupil dilation typically observed in normal aging. We used the simple procedure proposed by Allard, Wadlinger [25], who applied the following transformation to account for age differences in pupil diameter: ([minimum overall pupil diameter–current pupil diameter]/[maximum overall pupil diameter–minimum overall pupil diameter]). This transformation accounts for both age and individual differences and for performances on a given trial (e.g., the word ‘blue’ written in the color red) compared with a whole experimental condition (e.g., the interference condition on the Stroop task).

To summarize, pupil dilation is a physiological index of cognitive processing in humans [26,27,28]. Using pupillometry, research has reported increased pupil dilation in the interference condition of the Stroop task in younger adults, which may be attributed to the high cognitive load of this condition [8,9,10,11,12]. However, this experiment did not investigate aging. We thus assessed whether older adults would demonstrate increased pupil dilation when performing the interference condition of the Stroop task compared with the control conditions (i.e., color-naming and word-reading conditions). Using these procedures and a normalized measure to overcome the limited range of pupil dilation in normal aging, we expected increased pupil dilation in the interference condition of the Stroop task compared with the control conditions.

## 2. Method

### 2.1. Participants

The study included 44 older adults (24 women and 20 men; mean age = 65.32 years, *SD* = 4.71) and 44 younger ones (23 women and 21 men; *M* age = 22.03 years, *SD* = 3.73), who voluntarily participated in the study. The older participants were non-institutionalized and recruited in the local community. The younger subjects were predominantly graduate/undergraduate psychology students. Significant differences between groups were observed for age [*t*(86) = 47.79, *p* < 0.001] but not for gender [χ^2^(1, *N* = 88) = 0.05, *p* = 0.83]. Exclusion criteria were a history of learning, psychiatric, or neurological disorders. The older adults demonstrated a lower general cognitive ability than the younger ones, as assessed by the Montreal Cognitive Assessment [29] [*M* older adults = 27.31/30 points, *SD* = 1.12, *M* younger adults = 28.66/30 points, *SD* = 1.25, *t*(86) = 5.33, *p* < 0.001]. The original sample comprised 54 older adults and 51 younger ones: six older participants were excluded as they declared a history of psychiatric/neurological disorders and four were excluded as they performed below the norms (cutoff score of 26) on the Montreal Cognitive Assessment. Five younger adults were excluded as they declared a history of psychiatric/neurological disorders and two were excluded as they performed below the norms on the Montreal Cognitive Assessment. The final sample size (44 participants in each age group) was calculated a priori with the software G*Power [30]. We conducted a calculation for repeated measures (three within-subjects conditions on the Stroop task) in ANOVA tests, where the calculation was based on 95% power, an estimated probability of making Type I error as 0.05, and a medium effect size of 0.25 [31]. The study was conducted in accordance with the tenets of the Declaration of Helsinki, with a favorable opinion (number 20202-A02276-33) from the Committee for the Protection of Persons (the French national ethical board).

### 2.2. Procedures

The participants performed the Stroop task while wearing eye-tracking glasses (Pupil Lab) to record their pupil responses. This remote pupil-tracking system uses infrared illumination and has a gaze position accuracy of less than 0.1° and a 200 Hz sampling rate. Prior to starting the Stroop task, participants underwent a five-point calibration and validation process. The study took place in a quiet room at the psychology department of the University of Nantes, with closed blinds and a 60-watt fluorescent lamp providing the same level of lighting in both conditions to ensure that any differences in pupil dilation were not caused by differences in retinal illumination.

### 2.3. Stroop Task

The task consisted of three conditions: color naming, word reading, and color–word interference. This presentation order was kept constant for all participants (as in the typical Stroop task). Stimuli were presented using a laptop running Matlab and displayed on a separate 27-inch screen. In the color-naming subtest, participants had to name the color of 24 colored squares as fast and accurately as possible. In the word-reading subtest, they had to read 24 words written in black ink, all words naming colors, as fast and accurately as possible. In the word–color interference condition, they had to name the color of 24 color words presented in incongruously colored ink (for instance, the word ‘yellow’ was displayed in green), as fast and accurately as possible. We implemented 24 stimuli in each condition, as recommended in the paper and pencil French version of the Stroop task [32].

In the color-naming condition, each of the 24 trials began with the 1000 ms presentation of a black cross displayed at the center of a white screen. This cross was followed by a 200 × 200-pixel square appearing in the same position on a white screen. Each square was presented for 3000 ms and was either blue, green, red, or yellow. In the word-reading condition, each of the 24 trials began with the 1000 ms presentation of a black cross at the center of a grey screen. This cross was followed by the 3000 ms presentation of a color word (i.e., the French words for blue, green, red, or yellow) written in black in the same position on a white screen. In the interference condition, each of the 24 trials began with the 1000 ms presentation of a black cross at the center of a grey screen, which was followed by the 3000 ms presentation of a color word in incongruously colored ink in the same position on a white screen. In the three conditions, the beginning of pupillometric recording was synchronized with the appearance of the squares or words. The experimenter manually recorded response accuracy. Prior to the experiment, a training session was performed with four trials for each condition. This session was implemented as a warm-up and performances were not included in the analyses. 

Regarding dependent variables, we calculated the number of accurate responses: for instance, if a participant correctly named all of the squares in the color-naming condition, the score was 24 accurate responses (out of the 24 trials) and 0 inaccurate responses. Wrong responses, lack of responses, responses such as “I don’t know/hum” or other speech hesitations, were considered as inaccurate. Another dependent variable was pupil dilation. As mentioned in the Introduction, we applied a transformation procedure to determine the average rate of change in pupil dilation in each trial for older adults and younger adults, to account for age differences. This transformation procedure took into account the range of pupil diameters to calculate a ratio percentage in pupil dilation for a particular trial compared with the overall condition. This transformation was applied, using R program, within each subject and condition. The transformation was: ([minimum overall pupil diameter–current pupil diameter]/[maximum overall pupil diameter–minimum overall pupil diameter]). A higher ratio percentage score indicated a greater range of change in pupil dilation, and, consequently, an increased cognitive effort.

## 3. Results

We compared the accurate responses and pupil dilation with repeated measures ANOVA. The age group (older adults vs. younger adults) was the between-subject factor and condition (i.e., color-naming, word-reading, or interference condition) was the within-subject factor. Analyses were followed with *t*-tests comparisons for which we provided effect sizes by using Cohen’s *d* [31]: 0.20 = small, 0.50 = medium, 0.80 = large. For all tests, the level of significance was set at *p* ≤ 0.05.

### 3.1. Poor Performances in the Interference Condition in Older and Younger Adults

**Figure 1:**
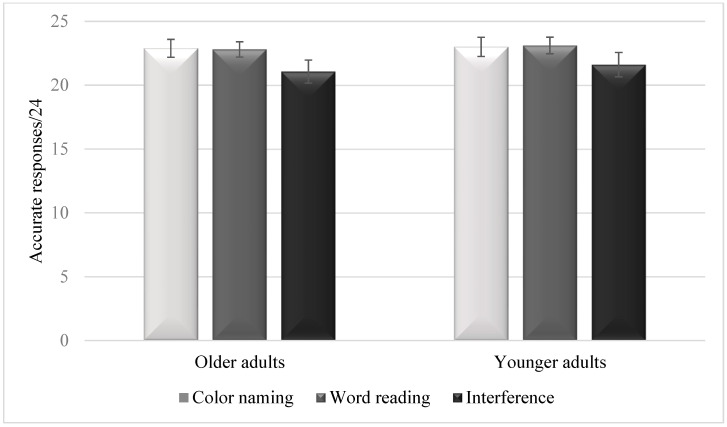
Means of accurate responses in the three conditions of the Stroop task in older adults and younger adults. Error bars are 95% within-subject confidence intervals.

Figure 1 summarizes the means of accurate responses in each condition and in each age group. The condition effect was significant, *F*(2, 172) = 24.39, *p* < 0.001, *η*^2^ = 0.22. Follow-up tests showed less accurate responses in the interference condition (*M* = 21.34, *SD* = 2.28) than in the color-naming condition (*M* = 22.94, *SD* = 1.30) [*t*(87) = 5.85, *p* < 0.001, Cohen’s *d* = 0.87] or the word-reading condition (*M* = 22.95, *SD* = 1.29) [*t*(87) = 5.39, *p* < 0.001, Cohen’s *d* = 0.81]. However, no significant differences were observed between the color-naming and the word-reading conditions [*t*(87) = 0.53, *p* = 0.96, Cohen’s *d* = 0.08]. The group effect was not significant, *F*(1, 86) = 2.53, *p* = 0.12, *η*^2^ = 0.03. The interaction between group and condition was not significant either, *F*(2, 172) = 0.33, *p* = 0.72, *η*^2^ = 0.004. 

### 3.2. Significant Pupil Dilation in the Interference Condition in Older and Younger Adults

**Figure 2:**
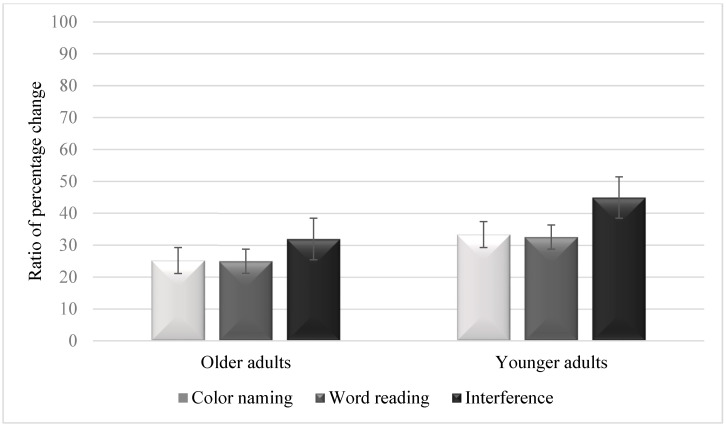
Ratios of percentage change in pupil dilation in the three conditions of the Stroop task in older adults and younger adults. Error bars are 95% within-subject confidence intervals. Note. Higher ratio percentage scores indicate greater pupil dilation.

Figure 2 summarizes the ratios of percentage change in pupil dilation in each condition and in each age group. The condition effect was significant, *F*(2, 172) = 54.25, *p* < 0.001, *η*^2^ = 0.38. Follow-up tests showed larger pupil dilation in the interference condition (*M* = 38.45, *SD* = 11.05) than in the color-naming condition (*M* = 29.25, *SD* = 9.59) [*t*(87) = 7.93, *p* < 0.001, Cohen’s *d* = 0.96] or the word-reading condition (*M* = 28.77, *SD* = 8.84) [*t*(87) = 7.89, *p* < 0.001, Cohen’s *d* = 0.88]. However, no significant differences were observed between the color-naming and the word-reading conditions [*t*(87) = 0.53, *p* = 0.96, Cohen’s *d* = 0.04]. The group effect was significant, *F*(1, 86) = 48.77, *p* < 0.001, *η*^2^ = 0.36, as older adults demonstrated lower pupil dilation (*M* = 27.37, *SD* = 11.21) than younger adults (*M* = 36.94, *SD* = 8.26). The interaction between group and condition was also significant, *F*(2, 172) = 4.04, *p* = 0.019, *η*^2^ = 0.04, as younger adults demonstrated larger pupil dilation across the three conditions.

The analysis further demonstrated lower pupil dilation in older adults than in younger adults in the color-naming condition [*t*(87) = 4.37, *p* < 0.001, Cohen’s *d* = 0.93], the word-reading condition [*t*(87) = 4.44, *p* < 0.001, Cohen’s *d* = 0.94], and the interference condition [*t*(87) = 8.61, *p* < 0.001, Cohen’s *d* = 1.84]. In older adults, larger pupil dilation was observed in the interference condition than in the color-naming condition [*t*(44) = 4.33, *p* < 0.001, Cohen’s *d* = 0.84] or the word-reading condition [*t*(44) = 4.37, *p* < 0.001, Cohen’s *d* = 0.85]. However, no significant differences were observed between the color-naming and the word-reading conditions [*t*(44) = 0.21, *p* = 0.83, Cohen’s *d* = 0.04]. In younger adults, larger pupil dilation was observed in the interference condition than in the color-naming condition [*t*(44) = 6.92, *p* < 0.001, Cohen’s *d* = 1.18] or the word-reading condition [*t*(44) = 7.05, *p* < 0.001, Cohen’s *d* = 1.20]. However, no significant differences were observed between the color-naming and the word-reading conditions [*t*(44) = 0.65, *p* = 0.52, Cohen’s *d* = 0.05]. 

## 4. Discussion

We investigated pupil dilation during the Stroop task when performed by older and younger adults. The results demonstrated fewer accurate responses in the interference condition than in the color-naming and conditions in both older and younger adults. Critically, larger pupil dilation was observed in the interference condition than in the color-naming and word-reading conditions in both older and younger adults. Difficulties in the interference condition were thus characterized by large pupil dilation in both populations compared with the color-naming and word-reading conditions. 

Both older and younger adults demonstrated fewer accurate responses in the interference condition than in the color-naming and word-reading conditions, thus replicating the well-known Stroop effect. The latter has been widely attributed to the cognitive costs of the interference condition, specifically to the need to inhibit the dominant tendency to read the words and name the color of the words instead. The interference condition thus induces a high cognitive load, which may explain the large pupil dilation observed in both younger and older adults. This assumption fits with the well-known relationship between pupil dilation and cognitive effort [13,14,15,33,34] and with previous research attributing pupil dilation in younger adults in the interference condition to its high cognitive load [8,9,10,11,12]. According to van der Wel and van Steenbergen [34], increases in task demands, as required in the interference condition of the Stroop task, typically lead to increases in pupil dilation. Our paper confirms this finding by demonstrating larger pupil dilation in the interference condition than in the color-naming or word-reading conditions in older adults. The high cognitive load of the interference condition may explain the large pupil dilation observed in older adults compared with the other relatively simple conditions.

Pupil dilation may thus be used as an indirect index of cognitive effort in aging. While the older adults in our study demonstrated similar performances in the Stroop task as those of the younger adults, their pupils dilated significantly during the interference task. Although older adults may demonstrate normal performances on some difficult cognitive tasks, the cognitive effort required in these tasks triggers pupil dilation. Can older adults demonstrate normal performances in the Stroop task? This issue was investigated by Ludwig, Borella [35], who assessed the performances of older and younger adults on two different formats of the Stroop task: a blocked paper-and-pencil format and a computerized item-by-item format. The results demonstrated age-related decline in the interference condition of the blocked paper-and-pencil format, but not in the item-by-item format. The authors suggested that the perceptual space in the blocked paper-and-pencil might make the stimuli less distinct, which in turn might increase the difficulties of older adults to select the item to respond to, particularly if the surrounding items have not been suppressed. They also suggested that the item-by-item format provides a “purer” measure of the ability of older adults to resist automatic and prepotent stimuli. Mirroring this suggestion, Rey-Mermet and Gade [36] conducted a meta-analysis on 176 studies assessing inhibitory tasks in aging. The results were against an inhibition deficit in aging for most tasks (e.g., Stroop and flanker tasks). However, older adults showed impaired inhibition in a few tasks (e.g., the go/no-go and stop-signal tasks). According to Rey-Mermet and Gade (2018), these findings call into question the hypothesis of inhibition decline in aging. These findings may explain why no significant differences were observed between older and younger adults regarding accurate responses on the Stroop task in our study. However, our research adds to this literature by demonstrating that the cognitive effort required for the interference condition can trigger increased pupil dilation in older adults. We thus demonstrate that pupil dilation closely responds to cognitive effort, at least for the interference condition of the Stroop task in normal aging.

Our findings also extend previous research on pupil dilation during emotional processing in normal aging. van Reekum, Urry [37] invited older adults to use a reappraisal strategy while exposed to a series of negative images. The results showed increased pupil dilation when participants were invited to use the reappraisal strategy compared with when they were invited to only look at the images. This dilation may be attributed to the enhanced cognitive control required for reappraisal strategies [38]. The effects of emotion on pupil dilation were also evaluated by research, demonstrating larger pupil dilation for positive than negative information [25,39], which is consistent with the socioemotional selectivity theory, suggesting that older adults prioritize positive information to maximize emotional well-being [40,41,42,43]. Overall, these studies support the notion that pupil dilation can provide insight into the cognitive and emotional processes involved in normal aging.

Another finding of our study was the lower pupil dilation in older than in younger adults, even with the normalized measure of pupil size applied. These results fit with the well-known effect of aging on pupil size, as it substantially decreases with aging. This phenomenon is commonly referred to as senile miosis [6,7]. This miosis has been attributed to the degeneration of the dilation muscle (the dilator pupillae) in the iris, inducing a linear decline in pupil size with aging [6,7]. This decline can be attributed to age-related changes in the locus coeruleus, a brain area involved in the production of noradrenaline [44,45]. Noradrenaline is a neuromodulator involved in cortical excitability [46], functional connectivity [47], and cognitive functioning [48,49]. Therefore, the lower pupil dilation observed in our older participants than in the younger ones is in line with senile miosis.

One potential limitation of this study is that we did not record reaction times for accurate responses during the Stroop task. Reaction time may provide more information about task performance, such as the processing speed. However, we were not able to record reaction time due to technical challenges related to verbal responses. Direct input between the voice recorder and the software for presenting the Stroop stimuli and recording pupil data was not possible. Another potential limitation of this study is the near-ceiling performances observed in both populations for accurate responses in the Stroop task. These high performances could mask any group differences in inhibition, which is in contrast with previous research demonstrating a decline in inhibitory processes with aging [1,2,50]. Furthermore, these ceiling performances could also be a factor contributing to the significant interaction observed between age and condition in relation to pupil dilation.

It would be worth investigating pupil dilation during performances on inhibition tasks in age-related diseases, such as Alzheimer’s Disease, as patients with Alzheimer’s Disease demonstrate a significant decline in inhibition [51,52,53].

To summarize, our study opens up novel avenues of research for evaluating cognitive functioning in aging using physiological measures. Integrating behavioral performance and pupil responses could further advance our understanding of the (dys)function of cognitive processing in aging. This research may also help to determine whether pupil dilation can predict cognitive performance in aging.

## Data Availability

Data is available upon request to the corresponding author.
